# The nexus between environmental tax and carbon emissions with the roles of environmental technology and financial development

**DOI:** 10.1371/journal.pone.0242412

**Published:** 2020-11-25

**Authors:** Muhammad Farhan Bashir, Benjiang MA, Muhammad Shahbaz, Zhilun Jiao

**Affiliations:** 1 Business School, Central South University, Changsha, Hunan, P. R. China; 2 School of Management and Economics, Beijing Institute of Technology, Beijing, P. R. China; 3 College of Economic and Social Development, Nankai University, Tianjin, P. R. China; Institute for Advanced Sustainability Studies, GERMANY

## Abstract

This study evaluates the impacts of renewable energy, environmental taxes, environmental technology, and financial development on carbon emissions in OECD economies from 1995 to 2015 by employing system-GMM and quantile regression approaches. Our empirical analysis indicates that environmental tax negatively affects carbon emissions; economic growth impedes environmental quality by increasing carbon emissions. Further, renewable energy consumption, environmental technology, and financial development improve environmental quality by decreasing carbon emissions. We suggest that changes in policymaking to promote sustainable economic growth and environmental quality should be prevent environmental degradation, but also inspire greater investments in new technologies and energy expertise in the renewables industry.

## 1. Introduction

Sustainable environmental quality has been emphasized as a vital part to successful sustainable economic development [1–3]. Many studies have suggested that lower carbon dioxide (CO_2_) emissions indicate improved environmental quality. Economic expansion activities are strongly attributed with environmental degradation, such as enormous increase in GHG emissions, particularly CO_2_ emissions [4, 5]. This increase subsequently contributes in environmental issues such as climate change and environmental degradation. In the year 2018, vigorous industrial and economic performances increase global energy consumption by 2.3%, which in turn escalated carbon emissions by 1.7%, i.e., 33.1 gigatonnes from 32.5 gigatonnes [6]. This trend is a threat to global environmental initiatives. And countries around the world have pledged to reduce carbon emissions through corrective actions to safeguard environmental quality [7]. This view is supported by the Paris Climate Agreement (COP21), which states that the global average atmospheric temperature will increase by two degrees Celsius in the absence of concrete environmental reforms. Reaching this threshold will severely affect every aspect of human life [8] and include the extinction of species, droughts, wildfires, higher sea levels, and decreased quantities of crops and fresh water. Subsequently, this will not only affect the quality of food and air, but also cause several health problems [9–11].

Carbon dioxide (CO_2_), is the major contributors of GHG emissions [12, 13]. And there is a general consensus among environmentalists [14] that past environmental initiatives, such as the Kyoto Protocol and United Nations Framework Convention on Climate Change are no longer sufficient, and new rules and regulations to promote renewable energy sources are required to improve environmental quality [15–17]. However, several studies have indicated that policymakers are facing challenges in balancing economic growth while reducing environmental degradation [18–20]. Thus, the greatest challenge faced by policymakers involves policy changes to produce reliable, less expensive energy sources to curb GHG emissions. From a practical perspective, several policy changes have been promoted to design environmental regulations that decisively impact CO_2_ emissions. The following three arguments are of particular interest from the economic, energy, and environmental perspectives: (1) the promotion of renewable energy; (2) the role of environmental taxes; and (3) the exploration of financial development. The first proposition aims to promote the usage of renewable energy sources to reduce CO_2_ emissions [21–23]. Concerning the next proposition, we argue that environmental taxes needs further attention as a policy tool as it will not only mitigate GHG emission but also promote renewable energy sources [24, 25]. Finally, several studies have proven that the financial sector encourages technological advancements carbon emissions mitigation [26, 27] as credit market is integral in promoting the renewable energy sector. Further, Kim and Park [27] established that debt and equity financing through credit market has accelerated growth of renewable energy sector [28].

Moreover, from a research perspective, previous studies have only investigated the role of financial development and renewable energy on CO_2_ emissions by using either panel or time-series data. To the best of our knowledge, no previous study has assessed the sub-components of financial development, environmental taxes, economic growth, environmental technologies, and renewable energy consumption on CO_2_ emissions. This paper provides three contributions to existing literature: (I) It investigates the impact of environmental taxes and environmental technology on CO_2_ emissions by considering financial development, economic growth, and renewable energy consumption in OECD economies. An examination of the OECD region is of particular interest, as most OECD economies are developed and have a technological advantage to fully utilize renewable energy sources. (II) Unlike previous studies, our empirical analysis incorporates financial intermediation, size of the financial system, and financial globalization to provide a more comprehensive narrative regarding financial development and environmental degradation. (III) We also apply system-GMM, pooled OLS and quantile regression methods for examining the relationship between carbon emissions and their determinants. Our empirical analysis reveals that implementing environmental taxes can assist in improving environmental quality, while environmental technology has negative association with carbon emissions. While financial development and renewable energy consumption decrease carbon emissions, economic growth increases them.

The remainder of this paper is organized as follows: Section II provides an overview of empirical literature. Section III presents our empirical methodology. Section IV discusses the empirical findings and results, while Section V provides policy implications, and Section VI concludes.

## 2. Literature review

We divide this literature review to discuss the nexus between CO_2_ emissions and each of the following: environmental taxes, environmental technology, renewable energy consumption, financial development and economic growth to provide detailed outlook of existing literature.

### 2.1 The environmental tax-CO_2_ emissions nexus

Environmental taxes are an efficient policy instrument to decrease GHG emissions [29, 30]. The concentration of CO_2_ negatively correlates with environmental tax reforms [31], as environmental taxes aim to tax carbon emissions as the most common sources of GHG emissions [32]. Tamura et al. [33] extended this discussion to suggest the environmental tax decrease the total carbon emissions as it contributes to higher fossil fuel prices, leading to lower fossil fuel demand. Barker et al. [34] reviewed the EU policies to mitigate carbon emissions to suggest that environmental taxes are more effective in reducing carbon emissions, if EU policy directives complement member state policies.

Although environmental taxes are generally aimed at carbon emissions, such taxes also consist of energy and fuel taxes. These are useful in achieving the environmental protection targets set by different environment efforts, such as the Kyoto Protocol and Paris Climate Agreement [35]. Many studies [36–38] have confirmed environmental taxes’ effectiveness, although some researchers suggest that environmental taxes have only modest impacts on GHG emissions [39]. Further, Vera and Sauma’s [40] analytical arguments determined that environmental taxes from 2014 to 2024 will only produce a 1% reduction in GHG emissions. Environmental taxes reduce carbon emissions and are instrumental in promoting renewable energy, and decrease energy demands through progressive energy efficiency [41]. Numerous studies have suggested various data analysis models to investigate the association between carbon emissions and environmental taxes, such as the CGE model, Leontief type input-output model, GCAM model and OSE 2000 model [35, 40–42]. Recently, Miceikiene et al. [43] investigated whether environmental taxes protect environmental quality, and noted that such taxes are prominent in improving environmental quality if innovations in the energy and environmental sectors are prioritized.

### 2.2 The environmental technology-CO_2_ emissions nexus

Research examining the association between environmental technology and carbon emissions has become popular, and can be divided into two main categories: environmental technology’s impact to reduce carbon emissions and promote cleaner energy sources. For example, Sun et al. [44] examined environmental patents’ impact on carbon emissions using Chinese provincial data, and contended that environmental technologies significantly decrease carbon emissions as advanced environmental technologies provide systems solutions. Weixian and Fang [45] observed a case in China by studying the development of environmental technologies, and suggested that environmental technologies decrease carbon emissions as environmental innovation’ positive influence in technological domains prevents environmental degradation. Similarly, Nesta et al. [46] suggested that the development of environmental technology is influenced by the adoption of renewable energy policies. They further noted that renewable energy policies are useful in promoting innovations in green technology, and are responsible for reducing carbon emissions. Kahouli [47] studied Mediterranean economies for the period spanning 1990 to 2016 to conclude that investments in technological developments exhibit an inverse relationship with carbon emissions; this strongly suggests that environmental technologies prevent environmental degradation as these countries significantly encouraged R&D investments into environmental technologies to reduce GHG emissions. Similarly, Fernández et al. [48] observed cases in China, the European Union, and the United States to conclude that research and development (R&D) expenditures in the European Union and United States were vital in reducing carbon emissions, although they had an inverse effect on the Chinese economy. These authors further suggested that R&D investments to develop environmental technologies would be appropriate measures in reducing carbon emissions. Further, Chen and Lei [49] explored the environmental-energy-growth nexus among a panel of 30 nations to conclude that environmental technologies significantly reduce carbon emissions. Subsequently, they suggested that countries with the most carbon emissions should further invest in environmental technologies to preserve the environment.

Alternatively, environmental technologies are also influential in developing and promoting green energy [50]. Generally, environmental technologies improve environmental quality and reduce carbon emissions by promoting renewable energy sources. For example, Meliciani [51] examined the association between R&D and patents for panel data from 27 countries to contend that R&D investments preserve environmental quality by encouraging investments in development of environmental patents. Sohag et al. [52] researched the Malaysian economy to examine the association between innovation, economic growth, and energy use from 1985 to 2012; their work revealed that technological innovations minimize the carbon footprint. They further suggested that state initiatives to promote public-private partnerships should also promote innovations in energy-efficient technologies. Shahbaz et al. [53] analyzed the carbon emission function of the French economy by considering the role of energy innovations. Their empirical results indicate that energy innovations decrease energy intensity, which consequently decreases carbon emissions and improves environmental quality. Shahbaz et al. [54] then applied a carbon emissions function in the Chinese context by considering vital role of private-public-partnership investments in the energy sector and energy innovations. They discovered that energy innovations decrease carbon emissions, which then improve environmental quality, it led them to believe that environmental innovations play a significant role in mitigation of GHG emissions.

### 2.3 The renewable energy-CO_2_ emissions nexus

Developments in renewable energy sources have sparked lively debates over the last two decades regarding their impacts on economic growth and the environment [55, 56]. This is because renewable energy restricts climate change by reducing GHG emissions [57]. The OECD’s [58] energy report suggested that investments in traditional energy are more carbon-intensive than those for renewable energy sources; hence, renewable energy’s bidirectional causal relationship with carbon emissions, trade openness and sustainable economic growth not only promotes green environmental reforms, it offers economic and energy-related benefits [59, 60].

The economic benefits of renewable energy include the diversification of a country’s energy portfolio; increased energy security; decreased outflows of foreign currency; and employment opportunities, as the renewables sector offers more employment opportunities than with non-renewable energy sources [61, 62]. Further, the adoption of renewable energy sources reduces the dependence on oil imports for countries with energy sectors heavily dependent on such imports [63]. However, this offers oil-exporting countries—such as those in the Organization of the Petroleum Exporting Countries (OPEC)—an opportunity for economic diversification [63]. Overall, past literature suggests that findings are inconclusive and results vary according to econometric techniques; the data series adopted, such as panel or time-series; economic attributes; and the chosen time frame [64].

Several studies have indicated a bidirectional causality exists between renewable energy consumption and CO_2_ emissions [65, 66]. Further, literature has well-documented renewable energy’s role in reducing carbon emissions and contributing to environmental quality [58, 67]. For example, Apergis et al. [68] employed health expenditures as an independent variable to conclude that renewable energy reduces carbon emissions; Ben Jebli et al. [69] also confirmed similar results. Recently, Charfeddine and Kahia [70] examined the relationship between renewable energy consumption and carbon emissions for the MENA region. Their empirical analysis indicated that carbon emissions respond negatively due to standard shock occurs in renewable energy consumption.

### 2.4 The financial development-CO_2_ emissions nexus

Generally, researchers strongly support the idea that economic growth is significantly supported by financial development [71, 72]. There is a general consensus that financial development serves as a pillar for economic growth, as it mobilizes savings, refines information for investment decisions, and is integral in capital allocation. And contributes in eliminating GHG emissions by allocating financial resources to developing technological advances [73]. These findings support the general perception that financial development is important in eliminating environmental degradation by allocating financial resources for such endeavors; moreover, it integrates stock markets and banks into the financial sector [74–76] as a funding source for renewable energy. The financial sector also supports R&D activities influences environmental quality by incentivizing green projects [77, 78]. The key attributes associated with a developed financial sector include the promotion of investment activities, fewer costs for borrowing funds, and decreased environmental pollutants by boosting the energy sector’s efficiency [77, 78]. However, higher manufacturing activities due to credit access results in environmental degradation and adversely impact environmental quality especially in emerging economies, where regulatory institutions need to adopt stringent approach to protect the environment [73].

Theoretically, a lack of consensus exists regarding how financial developments impact economic growth and carbon emissions. Many studies have articulated different causal relationships for carbon emissions, economic growth, and financial development. Nazir et al. [79] examined the impacts of financial development, trade, and urbanization on carbon emissions in BRICS (Brazil, Russia, China, India, and South Africa) economies to posit that financial development decreases carbon emissions mainly due to availability of financial credit environment protection technology. In contrast, Phong [80] investigated globalization, financial development, and environmental degradation among emerging economies and found that financial development resulted in higher carbon emissions, especially in the countries where energy production has higher dependence on fossil fuels. Bekhet et al. [81] empirically analyzed the relationship between financial development and carbon emissions in Gulf Cooperation Council economies from 1980 to 2012 to discover a unidirectional causality from financial development to carbon emissions. Shahbaz et al. [53] examined the relationship between financial development and carbon emissions by adding foreign direct investments in a carbon emissions function. The authors revealed that financial development positively directs the improving of environmental quality. Among Asia-Pacific countries, Zaidi et al. [82] also noted that financial development decreases carbon emissions as financial development impacts the environmental quality through capitalization, improved regulations and environmental technology.

### 2.5 Economic growth—CO_2_ emissions nexus

The GDP growth is one of primary macroeconomic factors for countries’ policy making [83] as reaching a desired growth rate is considered as main economic objectives. However, ecological and environmental costs cannot be ignored. Therefore, the economic growth-CO_2_ nexus has gained the attention of policy makers, practitioners and researchers in recent times. Some existing studies have suggested the existence of U-curved association between economic growth and CO_2_ emissions also known as EKC (Environmental Kuznets Curve) hypothesis. Selden and Song [84] and Grossman and Krueger [85] were among the pioneer studies to imply that economic growth contributes in environmental degradation initially, and after reaching a certain economic threshold, environmental quality improves. These findings contradicted Bashir et al. [86], who suggested that CO_2_ emissions increase parallel with economic growth.

Stern [87] reported that carbon emissions start to decrease after domestic economy reached a certain income level. Bengochea-Morancho and Martínez-Zarzoso [88] examined a panel of high- and low-income countries to reveal that carbon emissions and economic growth were positively and negatively related for high and low-income countries, respectively. Joseph [89] analyzed sub-Saharan economies through panel co-integration analysis to show that carbon emissions and economic growth have positive impact on one another. Alkhathlan and Javid [90] also reported positive association between GDP and carbon emissions in the developing economies and further suggested that electricity contributes in less environmental degradation than fossil fuel consumption. Likewise, Hamdi and Sbia [91] examined long-run association between economic output, energy consumption and carbon emissions in Gulf cooperation council countries to suggest that carbon emissions and economic growth have long-run directional causal association. In another attempt, Muftau [92] used co-integration technique to test long-run equilibrium between economic growth and carbon emissions. They suggested that in the long run N-shaped link between CO_2_ emissions and economic growth in the west African economies. Rahman and Kashem [93] investigated the causality association between industrial growth, energy consumption and carbon emissions to report the long run nexus between carbon emission and industrial growth in Bangladesh. Rahman [94] and Mbareki [95] also reported positive association between economic growth and carbon emissions for a panel of Asian economies and Tunisia, respectively. Though, Saidi and Hammami [96] in a comprehensive research attempt, investigated a panel of 58 countries to suggest that economic growth has negative association with carbon emissions in the long run.

## 3. Data and empirical modeling

### 3.1 Empirical modeling and data collection

This study examines the effects of environmental taxes, renewable energy, economic growth, environmental technology, and financial development on carbon emissions for 29 OECD economies for the period spanning 1995 to 2015, as per data availability. The list of OECD countries under consideration include Australia, Austria, Belgium, Canada, the Czech Republic, Denmark, Finland, France, Germany, Greece, Hungary, Ireland, Italy, Japan, South Korea, Luxembourg, Mexico, the Netherlands, New Zealand, Norway, Poland, Portugal, Slovakia, Spain, Sweden, Switzerland, Turkey, the United Kingdom, and the USA. The implementation of environmental taxes is part of an environmental regulation process for improving environmental quality toward attainable economic development. Using renewable energy during the production process produces far fewer (negligible) carbon emissions, which further improves environmental quality. Further, economic growth may affect carbon emissions through scale and technique effects. Specifically, economic growth impedes environmental quality if the scale effect dominates the technique effect; otherwise, economic growth improves environmental quality by decreasing carbon emissions if the technique effect is greater than scale effect. Environmental technologies, such as energy-efficient, environmentally protective innovations, may decrease carbon emissions and consequently improve environmental quality. Financial developments may involve the distribution of domestic credit to firms that apply energy-efficient technologies, and this may encourage firms’ energy innovations. This may also improve environmental quality by decreasing carbon emissions. This discussion allows us to empirically calculate carbon emissions as the following function:

$$CO2_{it}=\beta_{1}RE_{it}+\beta_{2}EG_{it}+\beta_{3}ET_{it}+\beta_{4}DET_{it}+\beta_{5}FD_{it}+\varepsilon_{t}$$ (1)

where *i* represents the country, and *t* represents time; *CO2*, *RE*, *EG*, *ET*, *DET*, and *FD* represent carbon emissions, renewable energy, economic growth, environmental taxation, the development of environmental technology, and financial developments, respectively; *β* and *ϵ* represent the estimator and error terms, respectively. The variables are all converted into a logarithm form to reduce bias in the empirical estimations. Including financial development components can further transform Eq (1) as follows:

$$CO2_{it}=\alpha_{1}RE_{it}+\alpha_{2}EG_{it}+\alpha_{3}ET_{it}+\alpha_{4}DET_{it}+\alpha_{5}FIS_{it}+\alpha_{6}SFS_{it}+\alpha_{7}FG_{it}+\varepsilon_{t}$$ (2)

where *FIE, SFS*, and *FG* denote the efficiency of financial intermediation, the financial system’s size, and financial globalization, respectively.

Several variables are selected for the empirical analysis. (1) Carbon emissions, as measured in metric kilograms per capita, are measured using data from British Petroleum’s Statistical Review of World Energy. (2) Renewable energy consumption is noted as the share of renewable energy to total final energy consumption in OECD economies, or specifically, world development indicators. (3) Economic growth is a measure of income level, or GDP values in constant 2010 US dollars (world development indicators). (4) Environmental taxes are indicated by the value of environmental taxes in constant 2010 US dollars, as per the OECD’s database. (5) Environmental technology is measured by the number of patents registered per annum as per the World Intellectual Property Organization. (6) Financial development is represented by the efficiency of financial intermediation, the financial system’s size, and financial globalization, where such efficiency determines the efficiency of indirect financing activities from stock and bond markets; the financial system’s size is indicated by the amount of direct financing from financial markets; and finally, financial globalization is measured by the amount of loans allocated by non-resident banks in the domestic economy. Empirical values for the efficiency of financial intermediation, the financial system’s size, and financial globalization are sourced from the International Monetary Fund’s Financial Structure Index. Financial development sub-components are part of our research hypothesis to investigate the role of financial development in integrating economic growth with the energy sector. Another plausible explanation is that an innovative financial structure triggers the energy sector, environmental innovation, and economic reforms to achieve desired economic goals [54]. The descriptive statistics of empirical dataset are presented in the Table 1.

**Table 1:** Descriptive statistics.

Variables	Mean	Std. Dev.	Min.	Max.	*p*1	*p*99	Skew.	Kurt.
CO_2_	5.056	1.347	2.083	8.676	2.201	8.651	0.381	2.932
GDP	10.371	0.645	8.786	11.626	8.95	11.561	-0.617	2.619
Tax	9.442	1.168	6.55	11.662	6.784	11.642	-0.064	2.475
Innovation	7.086	2.388	2.079	12.59	2.944	12.351	0.248	2.063
Financial Intermediation Efficiency	0.354	0.286	0	1.85	0	1.618	1.887	8.87
Financial System’s Size	1.105	0.566	0	3.671	0	3.318	1.162	6.724
Financial Globalization	0.392	0.384	0.012	2.198	0.016	1.806	2.044	7.733
Renewable Energy	14.093	13.258	0.444	60.188	0.7	59.415	1.616	5.434

### 3.2 Estimation strategy

#### 3.2.1 IPS and CIPS unit root tests

Following the model estimation, we performed numerical estimations by applying a cross-sectional dependence test as developed by Pesaran [97], as interdependence exists in the developed economies’ data due to global and economic integrations. Further, IPS and CIPS unit-root tests developed by Im, Pesaran, and Shin [98] examined the data series’ stability. After analyzing the data series’ validity and cross-sectional dependence, we examined the long-term associations in our study’s primary variables using co-integration tests from works by Kao [99], Pedroni [100, 101] and Westerlund [102]. The co-integration test developed by Kao [85] adopts a co-integrating vector, which is same across all panels; Pedroni [83, 86] allows panel-specific co-integrating vectors; and Westerlund [102] uses an error-correction model (ECM) to check whether the data requires error corrections.

#### 3.2.2 Quantile regression

After confirming co-integration in the data sample, we applied a panel quantile regression as introduced by Koenker and Bassett [103]. The quantile regression quantifies the heterogeneous effects of covariates through conditional quantiles of the dependent variable, and offers a better summary of centrality than different quantiles in the presence of asymmetry [104, 105].

$$y_{i}=x\prime_{i}\beta_{\theta }+\mu_{\theta i},0<\theta <1$$ (3)

$$Quant_{\theta }\left(y_{i}/x_{i}\right)=x_{i}\beta_{\theta }$$ (4)

where *x* represents the explanatory variables’ vector, *y* represents the explained variables; *μ* denotes the error term, with a distribution of the conditional quantile that equals zero. The dependent variable’s *θ*th quantile is *Quantθ*(*y_i_|x_i_*). Further, ${\hat{\beta }}_{\theta }$ is a regression estimator of the *θ* quantile and is solved using the following formula:

$$min\sum_{y_{i}\geq x\prime_{i}\beta }\theta \left|y_{i}-x_{i}\beta \right|+\sum_{y_{i}<x_{i}\beta }\left(1-\theta \right)y_{i}-x\prime_{i}\beta$$ (5)

Different parameters will be estimated when θ equals different values. We select several different quantiles to efficiently examine the complex relationship between environmental taxes and energy consumption (energy intensity), such as the 10th, 25th, 50th, 75th, and 90th quantiles.

#### 3.2.3 Pool OLS and system GMM

Moreover, we further ensure that our empirical results are consistent and not spurious by applying pooled OLS (with Driscol and Kray standard errors) and system-GMM methods. These are employed later to provide constant, efficient approximations in a regression in which the independent variables are not strictly exogenous [106]. The system-GMM is also considered as it (i) is suitable when empirical growth models are employed with fewer periods and relatively large number of countries, (ii) solves the problems of fixed effects and probable endogeneity among control variables, and (iii) provides more reliable and efficient empirical estimates than other analytical estimators. Thus, Eq (2) is transformed to empirically estimate the system GMM, as:

$$\begin{array}{l} CO2_{i,t}-CO2_{i,t-1}=\beta_{1}(RE_{i,t-\gamma }-RE_{i,t-2\gamma })+\beta_{2}(EG_{i,t-\gamma }-EG_{i,t-2\gamma })+\beta_{3}(ET_{i,t-\gamma }-ET_{i,t-2\gamma })+\beta_{4}(DET_{i,t-\gamma }-DET_{i,t-2\gamma })+\\ \beta_{5}(FIS_{i,t-\gamma }-FIS_{i,t-2\gamma })+\beta_{6}(SFS_{i,t-\gamma }-SFS_{i,t-2\gamma })+\beta_{7}(FG_{i,t-\gamma }-FG_{i,t-2\gamma })+(\mu_{i,t}-\mu_{i,t-}{}_{\gamma })+\varepsilon_{i,t-}{}_{\gamma } \end{array}$$ (6)

$$\begin{array}{l} \Delta CO2_{i,t}=\beta_{1}\Delta RE_{i,t-\gamma }+\beta_{2}\Delta EG_{i,t-\gamma }+\beta_{3}\Delta ET_{i,t-\gamma }+\beta_{4}\Delta DET_{i,t-\gamma }+\beta_{5}\Delta FIS_{i,t-\gamma }+\beta_{6}\Delta SFS_{i,t-\gamma }\\ +\beta_{7}\Delta FG_{i,t-\gamma }+\Delta \mu_{i,t} \end{array}$$ (7)

#### 3.2.4 Dumitrescu and Hurlin causality test

Additionally, Dumitrescu and Hurlin’s [107] test explores the causal relationship between the variables included in this study. This test adopts a vector autoregressive (VAR) framework to consider the data sample’s unobserved heterogeneity; it also determines the causal relationship with separate regressions for every cross-section of the variables.

## 4. Empirical results and discussion

Panel data methodology opens with the CD test to check for the existence of cross-sectional dependence, which is confirmed by the empirical results as reported in Table 2. Therefore, we reject the null hypothesis regarding the non-existence of cross-sectional independence. Subsequently, we further check the variables’ stationery properties in the presence of cross-sectional dependence and heterogeneity. In doing so, we applied IPS and CIPS unit-root tests to inspect the unit-root properties and reported in Table 3. Regarding the IPS unit-root test, economic growth and environmental taxes are stationary at level, but the rest of the variables are stationary at the first difference. The IPS unit-root test indicates that all the variables exhibit a mixed order of integration. Regarding the CIPS unit-root test, carbon emissions, renewable energy consumption, and environmental technology are stationary at level; however, the economic growth, environmental taxes, efficiency of financial intermediation, financial system’s size, and financial globalization are stationary at the first difference, or at I (1).

**Table 2:** Cross-sectional dependence test.

Variable	CD Test	*p*-value	Mean ρ	Mean *abs*(ρ)
Log CO_2_	22.274***	0.000	0.250	0.510
Renewable Energy	45.065***	0.000	0.500	0.720
Log GDP	79.060***	0.000	0.880	0.880
Log Tax	37.461***	0.000	0.420	0.570
Log Innovation	11.432***	0.000	0.130	0.550
Financial Intermediation Efficiency	16.819***	0.000	0.190	0.510
Financial System’s Size	2.009**	0.045	0.020	0.390
Financial Globalization	45.592***	0.000	0.510	0.630

Note: ***, **, and * denote significance at the 1%, 5%, and 10% levels, respectively.

**Table 3:** IPS and CIPS unit root analysis.

Variable	IPS Unit-Root Test		CIPS Unit-Root Test	
	Level	First Difference	Level	First Difference
CO_2_	-1.169	-4.746***	-3.006***	-4.601***
Renewable Energy	0.6579	-4.025***	-2.883**	-4.458***
GDP	-2.307***	-2.9519***	-1.785	-2.838**
Tax	-2.1162**	-3.954***	-2.595	-4.188***
Innovation	-1.916	-4.2960***	-2.918***	-4.137***
Financial Intermediation Efficiency	-1.0947	-4.1550***	-1.858	-3.651***
Financial System’s Size	-1.8960	-3.4631***	-2.099	-3.360***
Financial Globalization	-1.2735	-3.7173***	-0.940	-3.130***

Note: ***, **, and * denote significance at the 1%, 5%, and 10% levels, respectively.

We chose co-integration tests from works by Kao [99], Westerlund [102], and Pedroni [100, 101] to analyze any co-integration between the variables; Table 4 displays the empirical results. Most of our findings are statistically significant at the 1% level, which confirms the presence of co-integration between the variables. We note that carbon emissions and independent variables move in the long-term in the case of OECD countries for the sampled time period.

**Table 4:** Panel co-integration analysis.

Panel Co-Integration Analysis	*t*-statistics	*p*-value
**Pedroni’s (1999, 2004) Co-Integration**		
Panel Modified Phillips-Perron statistics	5.3546***	0.000
Panel Phillips-Perron statistics	-10.3588***	0.000
Panel ADF statistic	-11.4273***	0.000
**Kao’s (1999) Co-Integration**		
Modified Dickey-Fuller *t*-statistic	-3.0791***	0.0010
Dickey-Fuller *t*-statistic	-4.2455***	0.000
Augmented Dickey-Fuller *t*-statistic	-1.6185**	0.0528
Unadjusted, modified Dickey-Fuller *t*-statistic	-5.1012***	0.000
Unadjusted Dickey-Fuller *t*-statistic	-5.1359***	0.000
**Westerlund’s (2005) Co-Integration**		
Variance ratio	-1.3074*	0.0955

Note: ***, **, * denote statistical significance at the 1%, 5%, and 10% levels, respectively.

Our main econometric analysis involves system GMM and panel quantile-regression approaches; Table 5 reports the empirical results. The empirical findings indicate that renewable energy has negative association with CO_2_ emissions, mainly due to renewable energy’s ability to promote energy-efficiency. Furthermore, renewable energy sources act as a policy control in restricting the fossil fuel consumption in the energy mix of OECD countries. Danish et al. [3] investigated EKC hypothesis for renewable and non-renewable energy consumption in Pakistan to indicate that renewable energy has bi-directional association with CO_2_ emissions, meaning higher renewable energy consumption reduces carbon emissions in the long-run. Apergis and Payne [56, 66] investigated renewable energy, CO_2_ emissions, and fossil fuels prices’ association in Central and south American countries and found long-term positive association between GDP, carbon emissions, and real oil prices. Furthermore, feedback association between study variables indicated the importance of renewable energy consumption in mitigation of carbon emissions. Next, economic growth is positively associated with environmental degradation, which is an expected outcome as higher energy consumption results in energy consumption. Govindaraju and Tang [108] analyzed the association between carbon emissions and economic growth in India and China to demonstrate that carbon emissions and economic growth exhibit positive feedback effects in the long run. Bildirici and Bakirtas [109] and Talbi et al. [110] observed Tunisia, and Abbas and Choudhury [111] examined BRICS countries, to report that carbon emissions promote economic growth in emerging economies but leads to environmental degradation. Alkhathlan and Javid [112] also supported our findings by investigating the association between carbon emissions, oil consumption, and economic growth in Saudi Arabia to discover that economic growth has a positive relationship with carbon emissions.

**Table 5:** Panel system-GMM and quantile regression analyses.

Variables	System-GMM		Panel Quantile Regression	
	Coefficient	*t*-statistics	Coefficient	*t*-statistics
Renewable Energy	-0.0151***	-10.310	-0.0164***	-8.4400
GDP	0.2108***	-6.2500	0.2581***	-5.7600
Environmental Taxes	-0.7559***	39.6100	-0.7221***	28.4800
Environmental Innovation	-0.1911***	20.5500	-0.1794***	14.5200
Financial Intermediation Efficiency	0.1077*	1.5900	0.1533*	1.7000
Financial System’s Size	-0.1421***	-4.4100	-0.0334	-0.7800
Financial Globalization	-0.0288	-0.4900	-0.0879	-1.1200
Constant	-0.9283	-2.6900	-0.2591	-0.5600
Observations	580	580	580	580
Wald’s Chi^2^ / R^2^	6,354.54	-	0.7402	-
Auto Correlation Test	-	-	-	-
VIF test	3.57		3.72	
Year Effects	Yes	Yes		

Note: ***, **, and * denote significance at the 1%, 5%, and 10% levels, respectively.

Environmental taxes have a significant, negative relationship with carbon emissions, demonstrating that environmental taxes have proven to be a successful strategy against environmental degradation. However, we suggest that environmental taxes should be distributed over a longer time frame to allow businesses to adapt to environmental policies while also maintaining economic competitiveness [113]. Our findings support the existing economic literature that OECD environmental efforts play positive role in reducing GHG emissions [10, 80]. Environmental technological innovations also negatively impact carbon emissions [114] as it promotes sustainable economic growth while endorsing the adoption of technological innovations to protect the environment [115]. Huaman and Jun [116] suggested that technological patents are vital in decreasing future carbon emissions by encouraging technological advances in energy sector. Lee and Min [117] deployment of environmental technology is crucial in controlling climate change. Next, the efficiency of financial intermediation positively impacts CO_2_ emissions in the OECD countries. This finding supports the assumption that financial institutions with higher costs will lack the capacity for financing, while higher credit costs result in problems for firms to receive funds for projects that involve higher carbon emissions. Yue et al. [118] investigated nonlinear relationship between energy consumption and financial development indicators in transitional economies to report that higher banking costs make it difficult to acquire funding for carbon intensive projects in transitional countries.

The financial system’s size exhibits a negative relationship with carbon emissions. This connection is intuitive, the financial system’s size indicates activity in the stock and bond markets, which make it easier for institutions to raise capital through active monetary platforms. Hence, this negative relationship confirms that projects involving higher carbon emissions will experience more difficulty in raising funds through public platforms in OECD countries. Paramati et al. [119] and Kutan et al. [120] supported similar findings to argue that financial reforms in OECD regions means that majority of financial institutions favor renewable energy projects to prevent fossil fuel consumption and environmental degradation. Lastly, Financial globalization has a negative relationship with carbon emissions for the OECD region, implying that financial globalization will be beneficial in limiting carbon emissions. Spencer and Stevenson [121] the association between energy consumption and, banking and capital markets in the EU region to claim that effective mobilization of low-carbon financing have reduced CO_2_ emissions. Umamaheswaran and Rajiv [122] investigated the strategic importance of renewable energy sector in India and reported that a conducive regulatory, financial and policy framework will play critical role in addressing GHG emissions in Indian economy.

Numerical estimates of the pooled OLS and quantile regression’s extended form are applied to examine the empirical results’ robustness, as reported in Table 5. The empirical results as reported in Table 6 indicate that renewable energy consumption is negatively linked with carbon emissions. Economic growth has a positive, significant effect on carbon emissions, and the relationship between environmental taxes and carbon emissions is both negative and significant. Environmental technology improves environmental quality by decreasing carbon emissions. Financial intermediation efficiency has a positive impact, although it is only significant in medium quantiles. The strength of financial institutions regulates the levels of risk, with higher cost-income and cost-assets ratios, which will result in financial institutions’ vulnerability to market risks, and eventually deter them from projects with higher carbon emissions. The financial system’s size exhibits a negative relationship with carbon emissions, but this is significant in higher quantiles. This implies that financial reforms in the OECD region have created difficulty in raising funds that rely on conventional financial sources, and financial sources must be sought that involve higher costs. Financial globalization also decreases carbon emissions in low and medium quantiles; this suggests that financial globalization is important in defining the energy structure in transitional economies. Further, a higher integration of financial reforms results in fewer atmospheric carbon emissions.

**Table 6:** Quantile regression extended in a quantile analysis.

Variables	OLS	Q10	Q25	Q50	Q75	Q90
Renewable Energy	-0.0286***	-0.0169***	-0.0156***	-0.0164***	-0.0102***	-0.0130***
	(-24.29)	(-13.01)	(-14.44)	(-11.84)	(-6.96)	(-13.56)
GDP	0.466***	0.149***	0.242***	0.258***	0.301***	0.375***
	(-14.31)	(-3.98)	(-8.99)	(-8.00)	(-7.60)	(-10.69)
Tax	-0.00575	-0.752***	-0.724***	-0.722***	-0.848***	-0.929***
	(-0.37)	(-45.06)	(-51.76)	(-39.63)	(-37.69)	(-47.87)
Innovation	-0.0321***	-0.181***	-0.174***	-0.179***	-0.170***	-0.185***
	(-6.75)	(-25.31)	(-29.26)	(-20.16)	(-12.81)	(-15.29)
F.I.E.	0.0104	0.115*	0.242***	0.153*	0.0941	0.00819
	(-0.54)	(-2.27)	(-5.04)	(-2.4)	(-1.18)	(-0.15)
S.F.S.	-0.00307	-0.0522	-0.0695**	-0.0334	-0.314***	-0.336***
	(-0.38)	(-1.56)	(-2.85)	(-1.09)	(-7.92)	(-10.49)
F.G.	-0.0565***	-0.109*	-0.118**	-0.0879	-0.0237	-0.0165
	(-4.18)	(-1.98)	(-2.8)	(-1.59)	(-0.35)	(-0.33)
Constant	0.327	-1.962	-0.616	-0.259	-0.292	-0.0361
	-0.98	(-4.96)	(-2.30)	(-0.78)	(-0.67)	(-0.08)
VIF	4.07	3.97	3.24	3.01	4.59	5.75
Observations	609	609	609	609	609	609

Note: ***, **, and * denote significance at the 1%, 5%, and 10% levels; standard errors are noted in parentheses.

We then apply Dumitrescu and Hurlin’s [107] causality test to examine the causal relationship between carbon emissions and their determinants; Table 7 reports the empirical results. The empirical estimates demonstrate that a feedback effect exists between renewable energy and carbon emissions. Specifically, renewable energy creates a Granger-type causality with carbon emissions, and in response, carbon dioxide emissions create Granger-caused renewable energy. This empirical finding is consistent with work by Saint Akadiri et al. [123], who supported the presence of a bidirectional causality between renewable energy consumption and carbon emissions for EU-28 countries. A bidirectional causal relationship exists between economic growth and carbon emissions, which demonstrates that economic growth and carbon emissions are interdependent. This empirical evidence is similar to results by Long et al. [124] for China, and Bekhet et al. [81] for Gulf Co-Operation Council nations, as these authors also note a feedback effect between the variables. Environmental taxes also exhibit a Granger causality with carbon emissions, and consequently, carbon emissions create a Granger-type causality with carbon emissions -or feedback effects- which is also supported by Xiao et al. [125] and Wang et al. [126]. We also find that efficient financial intermediation and carbon emissions have a bidirectional causal relationship. Similarly, Hassine and Harrathi [127] also supported the presence of a bidirectional causality in the Gulf region. A feedback effect is also noted between the financial system’s size and carbon emissions. Such effects were also confirmed by Ayeche et al. [128] for European economies, and Işik et al. [129] for Greece. A bidirectional causality is found from financial globalization and carbon emissions, in that financial globalization has a Granger-type causality with carbon emissions, and vice versa.

**Table 7:** Dumitrescu and Hurlin granger causality analysis.

Null Hypothesis	W-Stat.	Zbar-Stat.	*p*-value
Carbon emissions (log CO_2_) causalities			
Economic growth does not cause carbon emissions.	3.2808***	8.6851	0.0000
Carbon emissions do not cause economic growth.	2.0250***	3.9033	0.0001
Environmental taxes do not cause carbon emissions.	2.0892***	4.1477	0.0000
Carbon emissions does not cause environmental taxes.	1.6033**	2.2972	0.0216
Innovation does not cause carbon emissions.	3.1343***	8.1273	0.0000
Carbon emissions do not cause innovation.	1.5413**	2.0611	0.0393
Financial intermediation efficiency does not cause carbon emissions.	3.2478***	8.5595	0.0000
Carbon emissions do not cause efficient financial intermediation.	2.7145***	6.5288	0.0000
The financial system’s size does not cause carbon emissions.	2.2841***	4.8897	0.0000
Carbon emissions do not impact the financial system’s size.	1.7658**	2.9161	0.0035
Financial globalization does not cause carbon emissions.	4.0040***	11.438	0.0000
Carbon emissions do not cause financial globalization.	3.6622***	10.137	0.0000
Renewable energy does not cause carbon emissions.	6.3923***	20.533	0.0000
Carbon emissions do not cause renewable energy.	2.6947***	6.4531	0.0000

**Note:** ***, **, and * denotes significance at the 1%, 5%, and 10% levels, respectively.

## 5. Conclusion, limitations, and research extension

Despite the introduction of environmental initiatives of Paris Agreement (COP21) and Kyoto Protocol, the global temperatures and CO_2_ emissions have reached record levels. To prevent environmental degradation and achieve sustainable development, developed countries such as OECD are introducing environmental reforms, using advanced technological innovations and promote renewable energy into energy mix. This offers us an interesting avenue to explore determinants of CO_2_ emissions. The current research has analyzed renewable energy, economic growth, environmental taxes, environmental technology, and the sub-components of financial development’s impact on carbon emissions in the OECD region. Our empirical findings reveal that carbon emissions exhibit a negative relationship with renewable energy sources, demonstrating the continuously improving environmental quality in OECD economies. Economic growth has a positive impact on carbon emissions, while environmental taxes and environmental technology have a negative relationship with carbon emissions. Financial intermediation efficiency exhibits positive and the size of the financial system and financial globalization have negative relationships with CO_2_ emissions. Therefore, financial reforms in OECD economies have been able to restrict carbon emissions and promote renewable energy sources.

Empirical findings of current research provide important policy implications. OECD policymakers must introduce timely and efficient energy policies to mitigate environmental problems by adopting low-carbon energy fuels. For this, we present multiple policy recommendations. Legislators should put greater emphasis on renewable energy consumption through policy management and energy efficiency. In order for sustainable economic growth, individual governments should determine the suitable form of clean energy sources. Additionally, to reduce carbon emissions and conserve energy consumption, we recommend to promote renewable energy consumption by ensuring energy security in large energy consumption sectors like residential, transport and manufacturing etc. Policymakers must also promulgate reforms in financial sector to ease the credit access towards renewable energy sector, which will enable industrial and residential units to substitute non-renewable energy quickly.

Also, authorities can maximize the environmental impact of technological advancements to promote environmental preservation efforts. In this regard, governments need to introduce environmental legislation to foster technological initiatives and iconological innovations. Technological innovations and green policies must overcome social and environmental issues while promoting sustainable economic growth. We would also encourage policy changes to set benchmarks to evaluate the role of financial development in promoting green technologies to improve environmental quality. Financial development builds market platform to allow businesses to foster profound synergies and share innovative technologies. Also, government policies should encourage environmental education and awareness to promote sustainable economic growth.

This study has the following limitations that could serve as directions for further research: First, we have used patents as a proxy for eco-innovation. Although environmental patents provide comprehensive evidence, but patents alone cannot explain eco-innovation, as the innovation process ranges from an idea and R&D to market applications. This missing information on eco-innovation undermines our research contributions, as environmental patents are only a part of new technologies; thus, future research can explore this point. Another research restriction involves considering the OECD as a whole, as environmental policies, carbon emissions, and innovations extensively vary among the OECD countries. Future country specific research in the OECD could offer country-specific suggestions and countermeasures.
